# Health-seeking behaviors and self-care practices of Dominican women with lymphoedema of the leg: implications for lymphoedema management programs

**DOI:** 10.1186/1475-2883-5-13

**Published:** 2006-12-22

**Authors:** Bobbie Person, David G Addiss, L Kay Bartholomew, Cecilia Meijer, Victor Pou, Bart van den Borne

**Affiliations:** 1 Centers for Disease Control and Prevention, National Center for Infectious Diseases, Office of Health Communication, Atlanta, Georgia, USA; 2 Centers for Disease Control and Prevention, National Center for Infectious Diseases, Division of Parasitic Diseases, Atlanta, Georgia, USA; 3 Center for Health Promotion and Prevention Research, School of Public Health, University of Texas Health Science Center at Houston, USA; 4 Instituto Dermatológico de Cirugia y Piel (IDCP), Santo Domingo, the Dominican Republic; 5 University of Maastricht, School of Health Promotion, Maastricht, the Netherlands

## Abstract

**Background:**

In the Dominican Republic, a Latin American country with filariasis-endemic areas, more than 63,000 people have lymphatic filariasis and more than 400,000 people are at risk of future infection. In this paper, we explore the health beliefs, health-seeking behaviors and self-care practices of women with lymphoedema in filariasis-endemic areas to better understand the needs of women when developing lymphoedema morbidity control programs.

**Methods:**

Qualitative data were collected through semi-structured interviews of 28 women, 3 focus group discussions with 28 women, field notes and photographs.

**Results:**

Women described exhaustive and expensive attempts at seeking a cure for their lymphoedema. Family members were influential in providing women with initial care seeking referrals to indigenous healers credited with influence over physical, mental, spiritual and supernatural properties of illness. When indigenous treatments proved to be ineffectual, the women sought care from trained healthcare providers. Most healthcare providers incorrectly diagnosed the edema, failed to adequately treat and meet the needs of women and were viewed as expensive. Most women resorted to self-prescribing injectable, oral, or topical antibiotics along with oral analgesics as a standard practice of self-care.

**Conclusion:**

Healthcare providers must understand a woman's cultural perspectives of illness, her natural networks of support and referral, her behavioural practices of care-seeking and self-care and the financial burden of seeking care. In the culture of the Dominican Republic family members and traditional healthcare providers are influential advisors on initial health-seeking behaviors and self-care practices. For this reason family-oriented interventions, support groups for women and their families, community education and training on simple, low cost lymphoedema management techniques for indigenous healers are viable ways to influence the early detection, diagnosis and treatment of women with lymphoedema. The extensive use of injectable, oral and topical antibiotics by indigenous healers and women without medical supervision suggests a need for health education messages related to the risks of such practices.

## Background

The global prevalence of lymphatic filariasis, a mosquito-transmitted parasitic disease, is vast [1]. In the Americas alone an estimated 8 million people are affected [2]. In the Dominican Republic more than 63,000 people have lymphatic filariasis and more than 400,000 people are at risk of future infection [3]. In 1998, a global initiative aimed at eradication of the disease by the year 2020 was established [4]. This initiative, the Global Programme to Eliminate Lymphatic Filariasis (GPELF) was built upon the twin pillars of interruption of transmission through mass drug administration and morbidity control [5]. The Dominican Republic has initiated both of these strategies in the filariasis-endemic areas of the country, which include parts of the rural southwest and the capital city, Santo Domingo. In different endemic regions researchers have begun to look more closely at the quality of life issues related to the behavioural, economic, and social consequences of lymphoedema to inform lymphoedema management programmes about the needs of those affected by this condition [6-11].

In Latin America, however few have examined the complex relationship between health-seeking behaviors, self-care practices and the cultural context of these behaviours among women who live with chronic manifestations of disease [12]. Lymphoedema management requires compliance with a prescribed, simple regime of self-care practices including leg hygiene, elevation, exercise, and self-examination with self-referral for appropriate medical care as needed [13,14]. Healthcare providers should understand a woman's cultural perspectives of illness, her natural networks of support and referral and behavioural practices of care seeking and self-management that she believes support her health [15]. Optimal care should also be informed by understanding the financial, psychological, and emotional costs associated with health-seeking that women face. The goals of this qualitative paper were 1) to explore specific health beliefs, health-seeking behaviors and self-care practices specific to women with lymphoedema in filariasis-endemic areas of the Dominican Republic, about which little is known; and 2) to provide recommendations for morbidity control programmes for lymphoedema management.

## Materials and methods

### Study Area

The Caribbean island of Hispaniola comprises two filariasis-endemic countries, the Republic of Haiti and the Dominican Republic [16]. Researchers in the Dominican Republic have documented lymphatic filariasis in the poor, densely populated urban neighbourhoods of the cities called *barrios *and in the poor rural communities associated with sugarcane production in the southern municipalities called *bateys *[17]. While there are no data specific to Dominican women, a study in 1998 documented transmission among school children in parts of both study areas, the Barrio La Cienaga in Santo Domingo, with a microfilaremia prevalence of 12.8% (29/227), and Barahona City, with a microfilaremia prevalence of 4.1% (10/243) [18].

### Qualitative Data Collection Methods

These data were collected as part of a larger study on Dominican women with lymphedema that explored the impact of disease on multiple domains of life, economic vulnerability, health seeking behaviors, and social support. Qualitative methods were used to gather data for this topic because little is known about the beliefs, values, norms, traditions, roles, experiences, and understanding of the disease process by women with lymphoedema in the Dominican Republic. Qualitative techniques are especially relevant in areas where there has been little previous research and there is a need for discovery, descriptive accounts, and an understanding of how people interpret and explain a social phenomenon or personal experience [19]. Data were collected through semi-structured interviews, focus group discussions (FGD), field notes, and photographs of women from January 2002 to October 2004 [20,21]. A semi-structured interview guide was used to encourage women to discuss their perceptions of illness, healthcare sought from indigenous healers and trained physicians, medical treatments, results of treatment, costs of treatments, and self-management of their lymphoedema. Questions were translated into Spanish, then pretested on a sample of local women with lymphoedema and modified to correspond with local dialect and cultural meanings before use in the interview guide. A bilingual research assistant back-translated the guide into English to ensure congruence with the original questions. Other women with lymphoedema participated in 3 focus groups. These groups discussed new and emerging ideas that arose from the in-depth interview data. Focus group data along with researcher notes served in triangulating the data. Questions in the FGD guide encouraged women to discuss in detail their attempts to seek a cure, types of traditional care sought, experiences of seeking care from trained physicians and their current regime of care.

We used modified precepts of grounded theory, an inductive method that elicits theory from the data, moving from the specific to the more general perspective [22]. Ideas emerging from the data guide gave the researcher new ideas to explore as they arose during the interview and across interviews. The constant comparisons of themes within and between interviews specifically informed and guided data analysis.

### Sampling and Recruitment

Researchers used a targeted sampling methodology to recruit women with lymphoedema of the leg (stages 3–7 based on the staging criteria of Dreyer et al) who lived in filariasis-endemic areas, participated in no prescribed lymphoedema management activities, or were inconsistent with lymphoedema management activities and agreed to be interviewed, audiotaped and photographed [13,23]. Based on the staging criteria of Dreyer, permanent (non-reversing) lymphoedema is considered Stage 3 if shallow skin folds are present; Stage 4 if there are protrusions or "knobs"; Stage 5 if deep skin folds are present; Stage 6, if "mossy lesions" are present; and Stage 7, inability of patient to perform activities of daily living [13].

In 2003, the Centro Para el Control de Enfermedades Tropicales (CENCET) conducted a lymphatic filariasis census in the southern, filarial-endemic region of the country prior to the administration of a programme of mass drug distribution for the interruption of transmission of disease [3]. The census data, collected by trained health workers, identified any woman with observable lymphoedema of the leg. Using this data public health officials referred women in the community with observable lymphoedema to local dermatology clinics associated with the Instituto Dermatológico de Cirugia y Piel (IDCP) in the coastal towns of Santo Domingo and Barahona. Dermatologists in each clinic screened and then referred women who met the study criteria to be interviewed prior to their being instructed in the care and management of their lymphoedema. Of approximately 70 women that were identified, 30 women meeting the screening criteria were referred to us for an in-depth interview. One woman refused to be interviewed and one interview tape was damaged. The 28 in-depth interviews, each approximately 2 to 3 hours long, took place in either the homes of the women or in the dermatology clinic before a lymphoedema management session

Twenty-eight other women participated in a 2-hour FGD at IDCP clinics; two such groups met in Santo Domingo and one group met in Barahona. Women who participated in the FGDs met the same criteria as did the women who were interviewed, although a few of the women in the FGD had previously received an educational session on lymphoedema management during prior clinic visits and were practicing the prescribed regime of care. Any woman coming into the dermatology clinic during the 2-week period prior to our visit, who had not been previously interviewed, was invited to participate in a FGD and the first eight women who agreed were accepted. If a woman cancelled, clinic staff called another woman from the clinic that met the screening criteria and asked her to participate. While women in the FGD generally did not know each other the similarity of their condition and their lives allowed them to bond during discussions which provided a safe space for expressing their feelings and experiences.

### Informed Consent

The institutional and ethical review boards of CENCET and the US Centers for Disease Control and Prevention (CDC) approved the study protocol, which required informed consent from each study participant.

### Data Management and Analysis

Semi-structured interviews and FGDs conducted in Spanish were audiotaped and transcribed verbatim. The transcripts were translated into English by a bilingual research assistant and then entered as a Microsoft Word^© ^document into ATLAS-ti to facilitate data coding, text searching and analysis [24]. Data was systematically examined for emerging codes, themes, and patterns. To ensure inter-coder agreement, two coders derived open coding schemes independently on a subset of interviews, then made comparisons to derive consensus on the coding strategy and repeated the coding independently on another subset of interviews to ensure consistency of the implementation of the coding strategy [25]. Themes related to the perceptions of illness, health-seeking behaviors associated with receiving care from indigenous or scientifically trained healthcare providers, treatments and treatment effects, self-management of disease, cost and cultural practices emerged as most salient to the women. Through axial coding and developing thematic tables and matrices, we were able to extract the direct quotes of women, categorize the quotes into codes and then the codes into categories and themes that women discussed, and then analyze any relationships we found among the data [22,25]. Consensus between the principal investigator and the research assistant was required in the interpretation and meaning of the chosen texts of women. When a meaning was not clear, we discussed the text segment and sought opinions with colleagues for clarification.

## Results

### Demographic Description

Sixty-seven percent of the 56 women in the study were over 40 years of age, with an average age of 50.5 and an age range of 26–80 years. Of the 56 women participating in the study, 40% had lymphoedema in each leg, 36% in just the left leg and 24% in just the right leg. Approximately 64% of women had severe lymphoedema, stages 4–6. Data from only the in-depth interviews of 28 women revealed that 46% were currently heads of households; 71% were married or in a free union or common-law marriage, 18% were widowed and 11% were single. Seventy-eight percent of the women could read and write. Ninety-four percent of women who were interviewed had sought care for chronic lymphoedema, with 96% seeking care from traditional providers and 90% seeking care from trained physicians at some time during the course of their disease. Seventy-two percent had sought care during episodes of acute adenolymphangitis. Of the other 28 women who participated in the FGDs, all had sought care for chronic lymphoedema and acute adenolymphangitis from both indigenous healers and scientifically trained physicians. Women in the study were from rural, semi-rural, and urban areas where mass drug distribution to interrupt transmission and lymphatic filariasis educational campaigns had taken place, but while many had taken the medications they usually did not associate the campaign with their condition.

## Local terminology and perceptions of causation

Few women in the in-depth interviews were able to answer questions correctly about the etiology of their lymphoedema and its association with lymphatic filariasis. Approximately half of the women in the FGDs talked about the association of lymphoedema with filariasis. Women in the FGDs also described seeing a recent campaign associated with mass drug distribution for the interruption of transmission of disease. Additionally, some of the women in the FGDs described receiving instruction in managing their lymphoedema, which included a discussion about transmission of lymphatic filariasis and the cause of lymphoedema.

The women in this study had several local names for the lymphoedema including, *erisipela *or *dicipela*. A few of the older women had heard the condition called *milk leg*. Women called an episode of acute adenolymphangitis a *crisis*, which was often preceded by a *seca*, or swelling of the inguinal lymph nodes. A *seca *might also be a large boil in the groin or upper thigh that often precedes or accompanies a *crisis*.

Spider bites, pregnancy, menopause, heredity, varicose veins, accidents and magical or supernatural events were common explanations for lymphoedema. Several women blamed individual children for their lymphoedema because of it's onset during their pregnancy. Many women with more severe lymphoedema attributed the onset of a *crisis *to having a wetness or a fungus between the toes, getting a cut on the leg, getting the foot wet or dirty, stubbing one's toe, working too hard or walking too far, or wearing underwear that is too tight and causes a *seca*. Women with stage 3, less severe lymphoedema, often reported not knowing what caused their *crisis *and often attributed it to supernatural causes such as having small birds in the leg or having a "hex" put on them.

## Healthcare-seeking behaviors

*"If I tell you all the people I have seen for this leg, it is not going to fit there on your paper," *said a woman with severe lymphoedema.

Women described exhaustive attempts at seeking treatments and a cure for their lymphoedema; they visited traditional herbalists, *ensalmadores *(specialized herbalists), *curanderos *(healers), *brujos *(witchdoctors), as well as trained physicians to try to find relief. Women sought care for a *crisis *of acute swelling, high fever, headaches, nausea, and pain, as well as the more chronic manifestations of the condition such as irreversible swelling and disfigurement, open wounds and boils, large skin folds, fungal infections and disagreeable odor. Despite the high prevalence of psychological distress described by most women, with a few women even having contemplated suicide at some point in their lives, no one described seeking care specifically for their psychological distress.

### Seeking Healthcare from Indigenous Healers

Most women described the first or second episode of acute adenolymphangitis as the impetus for first seeking care from an indigenous healer. Family members, acting as lay health advisors, served a critical role in positing the initial cause of lymphoedema as well as offering a referral to an indigenous healer. A few women told us that, while a family member may have taken them to an indigenous healer when they were children, they no longer sought them out because it conflicted with their Christian beliefs or they now viewed them as quacks. The indigenous healers women initially consulted to treat their lymphoedema included traditional herbalists, *ensalmadores*, *curanderos*, and *brujos*. Community members were also another source of advice on how to care for the leg or who to visit for a cure. Women most often described being *ensalmed. Ensalmadores *are people who have special techniques aimed at curing *erysipelas *or lymphoedema, including recitation of incantations and prayers to defeat supernatural powers that cause the condition, as well as the application of herbal preparations to the leg. The women also visited *curanderos *who addressed physical, spiritual, and mental anguish by consulting with saints in order to see which herbs, roots and various home cures to employ to dispel bad magic. Traditional *herbalists *prescribed bottles of liquid steeped with special herbs to drink, rub on the leg, and/or use as an enema as treatments for chronic lymphoedema. *Brujos *were most commonly used to rid the body of malevolent spirits that at times can take over the individual being treated. If one wanted to remove a "hex" or remove a spell they would visit a *brujo*. Several women described traveling to Haiti to seek a cure. Several women said that it was well known that this condition is prevalent in Haiti and the indigenous healers have more experience removing spells or bad magic. Women who had sought care from indigenous healers in Haiti were older, presented with the severest lymphoedema and had at some time in their life lived near the Haitian border.

Women described more than 65 treatments for lymphoedema of the leg suggested by indigenous healers, family members and friends. Recommended herbal remedies included prepared teas using local herbs and leaves, prepared creams, poultices of papaya and leaves, and the application of *higuereta*, a derivative of the castor bean, to the leg. Women also described using leeches, pricking the leg to drain it, soaking it in hot water, and pouring kerosene on the leg. Other treatments included sweeping the leg with a small broom, burying the leg in the ground, walking in salt water in the sea and rubbing the belly of a frog across the leg. *Ensalmadores*, *curanderos*, and *brujos *often prescribed injectable medications bought at the local pharmacy to supplement their traditional treatments for lymphoedema. A middle-aged woman said,

"When the lady ensalmed me the first time, she gave me some cream to put on my leg that night and the swelling of the leg got smaller because it was very affected. In addition, she had me take a penicillin injection. It cost twenty-five peso. She injected it in to my backside."

Women described some of the traditional treatments as having benefits or at least doing no harm. Poultices, such as papaya, and teas offered women some relief during a *crisis *when the leg was very hot and swollen and women experiencing severe headaches and nausea. However, many traditional treatments provided no relief for most women and some, such as pouring kerosene on the leg or attempting to drain the leg, exacerbated their condition.

*"My family wanted me to be cured, so my mother took me to see many brujos and healers. I almost died because of one of those old men," *said an older woman with severe lymphoedema.

The cost of traditional treatments ranged from no cost to substantial sums. A friend of the family might *ensalm *a woman for no money but barter a bottle of whiskey, a chicken, or another commodity. The women cited the cost of a single visit to an indigenous healer as ranging from 100–500 pesos ($2–10 US Dollars) and herbal preparations, teas, creams, and other items were an additional expense. Injections bought at a local pharmacy were relatively inexpensive and ranged from 15–125 pesos ($.20–2.50 USD). The women described the need for multiple sessions with multiple healers and the cost for these sessions ranged from 1,500–5,000 pesos ($30–100 USD). A woman seeking a cure said,

*"I have gone to so many people and spent so much money with this illness. It has taken away from me more than 60,000 pesos *($1,600 USD)*, and I have not seen any improvement."*

Women often required financial assistance from family members to pay their treatment costs.

We had informal discussions with one *ensalmadore *and one *curandero *neither of which would discuss in-depth their healing techniques but did share some general information with us. The *ensalmadore *was well known in the community. He described a general regime of hygiene and elevation that would be in keeping with recommended prescribed leg hygiene and elevation. Additionally, the *ensalmadore *described ritual prayers and conducted special incantations over the leg while applying traditional herbs to the leg. He also told us that when a woman's leg got very, very big the swelling would never go away and then it would then be in God's hands. The *curandero *suggested that a woman dig a hole in the ground and bury her leg in dirt on a regularly prescribed timeline. The woman would then have to bring an offering and pray to a specific religious saint every Friday. The *curandero *also prescribed specific tonics for enemas. The *ensalmadore *and the *curandero *required a payment of money and other goods for their treatments.

### Seeking Healthcare from Trained Physicians

Some women described seeking care from a medically trained physician and an indigenous healer at the same time. However, most women described seeking out trained physicians only after their attempts to treat their lymphoedema with indigenous treatments or home remedies failed. The women in our study described exhaustive attempts to find a physician who could treat their condition. A woman with lymphoedema for more than 10 years said,

"I am so tired and I have spent so much on doctors and more doctors. Many doctors have treated me but no one has helped. They just take my money."

Women sought care from a variety of physicians including those trained as general practitioners, cardiovascular surgeons, orthopedic surgeons, oncologists, acupuncturists, chiropractors, and dermatologists. Some physicians ran tests for lymphatic filariasis but when the tests came back negative they told the women that they did not know the cause of their lymphoedema. Many physicians incorrectly diagnosed the condition as varicose veins, cancer of the leg and afflictions of the spine. Physicians prescribed diuretics, analgesics, antipyretics, anti-inflammatories and antibiotics including ampicillin and penicillin (usually as an injectable). Physicians rarely gave women adequate explanations as to the cause of their lymphoedema or the purpose of their medications. However, many women saw positive changes when using antibiotics. Several women we interviewed had surgery to "unclog" their veins and reduce the size of their legs. The results left women with severe scarring and further disfigurement with no impact on their chronic lymphoedema or acute episodes of adenolymphangitis. In addition, the cost was substantial (up to $6,000 USD). Two women reported being told by their physician that their leg should be amputated, with one physician telling a woman that the lymphoedema was the result of cancer of the leg. Physicians rarely asked if women were receiving care from any indigenous healers and if women self-disclosed that information they were often ridiculed by the physician.

Women who saw no effect from the prescribed ointments, creams and pills their physicians prescribed were often distraught over the cost of care and the burden it caused their families. Most women agreed that antibiotic injections were the most successful treatment to stave off or treat a *crisis*. Women, individually and in the focus groups, described injections of antibiotics as the best treatment for boils, abscesses and other wounds on the leg. However, they expressed disappointment that antibiotics did not provide a cure for their chronic condition. Almost two-thirds of the women described buying injectable antibiotics from local pharmacies and self-injecting or having a family member or neighbor inject them without physician supervision. One woman who had recently had an episode of acute adenolymphangitis said,

"*The doctor solves everything with a prescription and I know he will give me a penicillin injection. So now I just do it myself. I don't go to the doctor anymore."*

Similar to the cost of traditional treatments, physician fees and medications ranged from no cost to substantial sums. Social security welfare visits were sometimes free and occasionally a woman would get a reduced cost because one of her family members worked at a healthcare facility. One focus group participant said,

"They gave me a reduced cost because my husband worked there. The visits to the hospital came out to be about 4,000 pesos ($80 USD) each."

Consultations with private physicians and clinics ranged from 200–1,000 pesos ($4–20 USD) and often required multiple visits with medical tests adding to their fees. Women described their medicines as expensive, ranging from 150–3,600 pesos ($3–72 USD). Injections bought at a local pharmacy were less inexpensive than those purchased from a physician. A woman with severe lymphoedema talked about the costs of her medications:

"I have to inject myself again with penicillin and buy a pain reliever. It ends up costing me 2,000–3,000 pesos to buy from the doctor. It's much, much cheaper to just buy the medicine myself."

Several women in the FGDs were currently participating in a lymphoedema management programme of prescribed hygiene, exercise and elevation with medications as needed. These women described positive interactions with dermatologists trained in simple, low cost lymphoedema management techniques. They described feeling better informed and more hopeful because physicians and support staff showed more concern for them. These women were pleased with the care they were receiving and were able to describe the cause of lymphatic filariasis, the cause of lymphoedema and techniques to care for their leg on a daily basis as well as during a *crisis*. Women were pleased with the healing of skin lesions and reduction of *crisis *but still disappointed at the lack of a cure.

## Discussion

In the Dominican Republic, as in other Caribbean and Latin American cultures, traditional healthcare providers and indigenous healers are an integral part of physical, mental, and spiritual healthcare for many people [26,27]. Researchers estimate that more than 3,000 indigenous healers, along with 16,000 physicians, practice in the Dominican Republic [28,29]. Currently, the extent and significance of treatment of lymphoedema by indigenous healers, as well as by trained physicians, are poorly documented. Indigenous healthcare beliefs and practices are the result of settlement of the country by Spanish explorers and later by the tribal customs and beliefs of African slaves [30]. This integration of cultures created a mixture of Catholicism, mysticism and other magico-religious means for dealing with life, illness, and death.

Findings from this study indicate that family, friends, and strong cultural beliefs influence women's explanatory model of illness (EMS) [31]. Women's EMS significantly influenced their health-seeking behaviors. A personal EMS is distinct from general beliefs about illness and health due to the specificity to explain a particular illness event. An EMS is the way a person perceives, mentally represents and culturally defines the episode of illness including the cause, symptoms and explanations for illness, diagnosis, treatments, and cultural role of the ill person [31]. A woman's EMS often guides her outcome expectations, as well as her coping strategies and health-seeking behaviors within a socio-cultural context and can change over time as we see in our findings. The reality that lymphoedema is culturally ingrained in the Dominican Republic is evident by the presence of *ensalmadores *who are described as having special techniques for curing *erysipelas *or lymphoedema.

Essential to any successful lymphoedema management programme is the understanding of the specific factors by which women interpret and place their illness experiences. The Dominican women in this study often had plausible explanations for their initial episodes of illness and solicited culturally relevant care and coping strategies from family, friends and recommended indigenous healers. Similar to findings from other countries, the current cultural paradigm of seeking indigenous healers as a first-line treatment often resulted in not only a significant delay in appropriate medical treatment but also potentially harmful treatments that could exacerbate the condition [32]. Researchers in cultural studies suggest that incorporating interventions within a cultural paradigm is more successful than trying to change a cultural paradigm altogether [15]. This theory suggests that providing lymphoedema management training or outreach to indigenous healers such as *ensalmadores*, as well as encouraging interactions of those individuals with more medically trained healthcare providers could create a vital mechanism for early treatment and referral. Previous public health efforts in Mexico, Ghana, Bangladesh, Zimbabwe and Swaziland have proposed that indigenous healers be incorporated into health systems in various ways, adapting to the specific features of their cultural practice and existing medical facilities and regulations [33-36]. Some developing countries are even encouraging regulation and licensing of traditional and indigenous healers and documentation of herbal preparations [36].

Reports from women, that many trained physicians often incorrectly diagnosed and mismanaged lymphoedema, suggests the need for continuing education for practicing physicians and other healthcare providers, as well as incorporation of diagnostic and treatment guidelines into the medical education curriculum. If lymphoedema management programmes are to be successful the need for communication between healthcare provider and patient is essential. Healthcare providers should give clear explanations about factors contributing to the disease, as well as the treatment regime and progressive nature of the condition. It is critical for women to perform self-examinations of the leg and identify potential complications needing a physician's care. When queried, women preferred simple visual instructions with photographs over just written instructions or written instructions with cartoon-like illustrations. A simple pocket-size photographic catalogue of possible conditions such as fungal infections, abscesses and boils could show women what to look for during their self-examinations and then how to treat or self-refer to appropriate healthcare providers.

The pervasive use of family members for emotional and financial support suggests that a family-oriented lymphoedema management intervention could be effective. Educating family members in the disease process, training them to manage lymphoedema and encouraging them to provide emotional support for women who have this condition could maximize limited programme resources. Overall cost of care places a significant burden on the individual and family members. Family-oriented interventions could encourage physical and psychological support in a more structured manner, a method that has worked with other chronic illnesses and allow judicious use of family resources [37].

Researchers studying the bio-behavioural responses of women to stress now posit that women respond to stress differently than men and characterize this response as "tend-and-befriend" [38]. They assert that affiliation with others and seeking social contact during stress and illness is an adaptive response with an underlying biochemical basis. In addition, research suggests that support by those who suffer with the same condition is especially valuable because they are seen as more creditable sources of information and there is little shame or stigma in sharing difficulties related to lymphoedema [39]. This is in-keeping with previous research which shows that support groups with other women can be a viable tool in providing psycho-educational opportunities with skill building sessions for low cost self-care, reinforcement for self-management efforts as well as emotional support and increased social connectedness for many women [6]. Women in our focus group sessions, who did not know each other prior to the focus group, asked when they could meet again because they shared a sense of connectedness with other women in the group. Additionally, family support groups could be an important strategy for caretakers of those with lymphoedema.

Self-management with antibiotics is a risky practice described by many women in this study. Women do not know what type of infection or other condition they may have, nor if they are injecting or ingesting an appropriate antibiotic to treat their condition. The global appearance of antimicrobial resistance is a multifaceted problem created by the convergence of many factors, including the use and misuse of antibiotics [40]. The extensive use of injectable and topical antibiotics by indigenous healers and women without medical supervision suggests a need for treating physicians to educate their patients on the need to follow medically supervised recommendations and to discuss potential risks of unsupervised antibiotic use with them. Indiscriminate use of antibiotics could contribute to antibiotic resistance.

## Conclusion

Similar to other countries, in the culture of the Dominican Republic, traditional healthcare providers, and indigenous healers are often an integral part of physical, mental, and spiritual healthcare for many people. Healthcare providers must understand a woman's cultural perspectives of illness, her natural networks of support and referral, her behavioural practices of care-seeking and self-care that she believes support her health and the financial burden of seeking care. Training both indigenous healers, considered as lay health advisors by women, along with physicians in simple, low-cost self-care regimes could increase the early detection, diagnosis, and treatment of lymphoedema among women in filariasis-endemic areas of the Dominican Republic. Family-oriented interventions and support groups for women and their families are viable ways to provide psycho-educational opportunities with skill building sessions, reinforcement for self-management efforts and emotional support for women. The often indiscriminate use of injectable antibiotics without medical supervision suggests a need for health education messages about this topic for women during lymphoedema management interventions. While these findings are specific to women in the Dominican Republic the methodology of inquiry into local cultural practices and beliefs to tailor interventions are not only applicable but recommended for other countries with lymphoedema management programmes.

## Ethical Approval

Institutional and ethical review boards from CDC and CENCET approved this study. Informed consent was obtained from every participant.

## Authors' contributions

BP conceived the study, coordinated the study, and developed the study instrument, collected data, entered data, conducted data analysis and drafted the manuscript. DA assisted in the design of the study, contributed to the study instrument, provided technical assistance throughout the course of the study and provided input into the writing of the manuscript. BB assisted in the design of the study and the study instrument, and assisted in data analysis and the writing of the manuscript. LKB assisted in the design of the study and the study instrument and contributed to the writing of the manuscript. VP assisted in conception and study design, recruited women for the study and reviewed the manuscript for intellectual content. CM assisted in translation during fieldwork, data collection, data entry and data analysis. All authors read and approved the final manuscript.

## Conflict of Interest

The author(s) declare that they have no competing interests.

## Disclaimer

The opinions or assertions contained in this manuscript are the private ones of the authors and are not to be construed as official or reflecting the view of the US Public Health Service or Department of Health and Human Services. Use of trade names is for identification only and does not imply endorsement by the US Public Health Service or Department of Health and Human Services.
